# Summation-based Private Segmented Membership Test from Threshold-Fully Homomorphic Encryption

**DOI:** 10.56553/popets-2024-0114

**Published:** 2024

**Authors:** Nirajan Koirala, Jonathan Takeshita, Jeremy Stevens, Taeho Jung

**Affiliations:** University of Notre Dame, Notre Dame, Indiana, USA; University of Notre Dame, Notre Dame, Indiana, USA; University of Notre Dame, Notre Dame, Indiana, USA; University of Notre Dame, Notre Dame, Indiana, USA

**Keywords:** Multi-party private set intersection, Private membership test, Fully homomorphic encryption

## Abstract

In many real-world scenarios, there are cases where a client wishes to check if a data element they hold is included in a set segmented across a large number of data holders. To protect user privacy, the client’s query and the data holders’ sets should remain encrypted throughout the whole process. Prior work on Private Set Intersection (PSI), Multi-Party PSI (MPSI), Private Membership Test (PMT), and Oblivious RAM (ORAM) falls short in this scenario in many ways. They either require data holders to possess the sets in plaintext, incur prohibitively high latency for aggregating results from a large number of data holders, leak the information about the party holding the intersection element, or induce a high false positive.

This paper introduces the primitive of a Private Segmented Membership Test (PSMT). We give a basic construction of a protocol to solve PSMT using a threshold variant of approximate-arithmetic homomorphic encryption and show how to overcome existing challenges to construct a PSMT protocol without leaking information about the party holding the intersection element or false positives for a large number of data holders ensuring IND-CPA^*D*^ security. Our novel approach is superior to existing state-of-the-art approaches in scalability with regard to the number of supported data holders. This is enabled by a novel summation-based homomorphic membership check rather than a product-based one, as well as various novel ideas addressing technical challenges. Our PSMT protocol supports many more parties (up to 4096 in experiments) compared to prior related work that supports only around 100 parties efficiently. Our experimental evaluation shows that our method’s aggregation of results from data holders can run in 92.5s for 1024 data holders and a set size of 2^25^, and our method’s overhead increases very slowly with the increasing number of senders. We also compare our PSMT protocol to other state-of-the-art PSI and MPSI protocols and discuss our improvements in usability with a better privacy model and a larger number of parties.

## 1 INTRODUCTION

Privacy concerns often limit the collaboration of entities in the case where each entity has private data that must be shared for joint usage. In many cases, the private data is generated and stored in a distributed manner, and methods of sharing data privately in such scenarios open avenues for new applications. There are many real-world scenarios where this problem needs to be solved.

One example of this is the case where federal tax authorities want to learn whether any suspected tax evaders maintain accounts in both domestic and foreign banks that might be under scrutiny and, only if so, obtain their account records and details. The banks’ locations in different jurisdictions prohibit the disclosure of account holders, and the tax authorities cannot openly divulge their list of suspects. Institutions under such scrutiny want to collaborate anonymously to avoid any bad publicity for being linked to a tax fraud operation. In many cases, these institutions are willing to collaborate with the tax authorities [61], and they themselves also wish to exercise rigorous scrutiny when extending loans to new and existing customers to mitigate potential risks. However, many financial privacy laws [85] prohibit banks from revealing customer data to third parties without consent. There are currently over 4,700 FDIC-insured banks in the United States. When a customer applies for a loan with one of them, collaboration and sharing of existing fraud lists can make the decision process significantly more trustworthy. In such cases, banks do not wish to share their private data on fradulent activities, and they do not even wish to disclose whether a queried customer is on their “fraud watchlist” due to various privacy and legal concerns. Such a secure membership query scenario could also include credit card companies, tax-collection agencies, and similar entities, necessitating the involvement of many parties in the decision-making process. A data-sharing protocol that allows queriers to learn only whether a queried entity exists in distributed fraud lists would help such institutions examine a person’s credibility beyond the nation.

As another example, many government agencies (e.g., FBI, CIA) maintain sensitive lists of secret agents or watchlists distributed across its multiple divisions and branches. Data sharing for identity verification, background checks, security clearance, or watchlist screening requires many government entities to work together. For example, a querier may want to verify an individual’s presence in these entities’ databases without inferring any membership details to verify the identity or the background. Doing so over distributed databases is nontrivial due to the challenges of sharing personally identifiable information (PII). It is desirable and even imperative that the records be stored in encrypted forms across a large number of distributed servers such that each record is under strict security/privacy protection (e.g., DHS Use Cases [86]). Similarly, the auto and medical insurance industries involve highly distributed records, and a protocol to query such databases can minimize risks.

**Figure 1:**
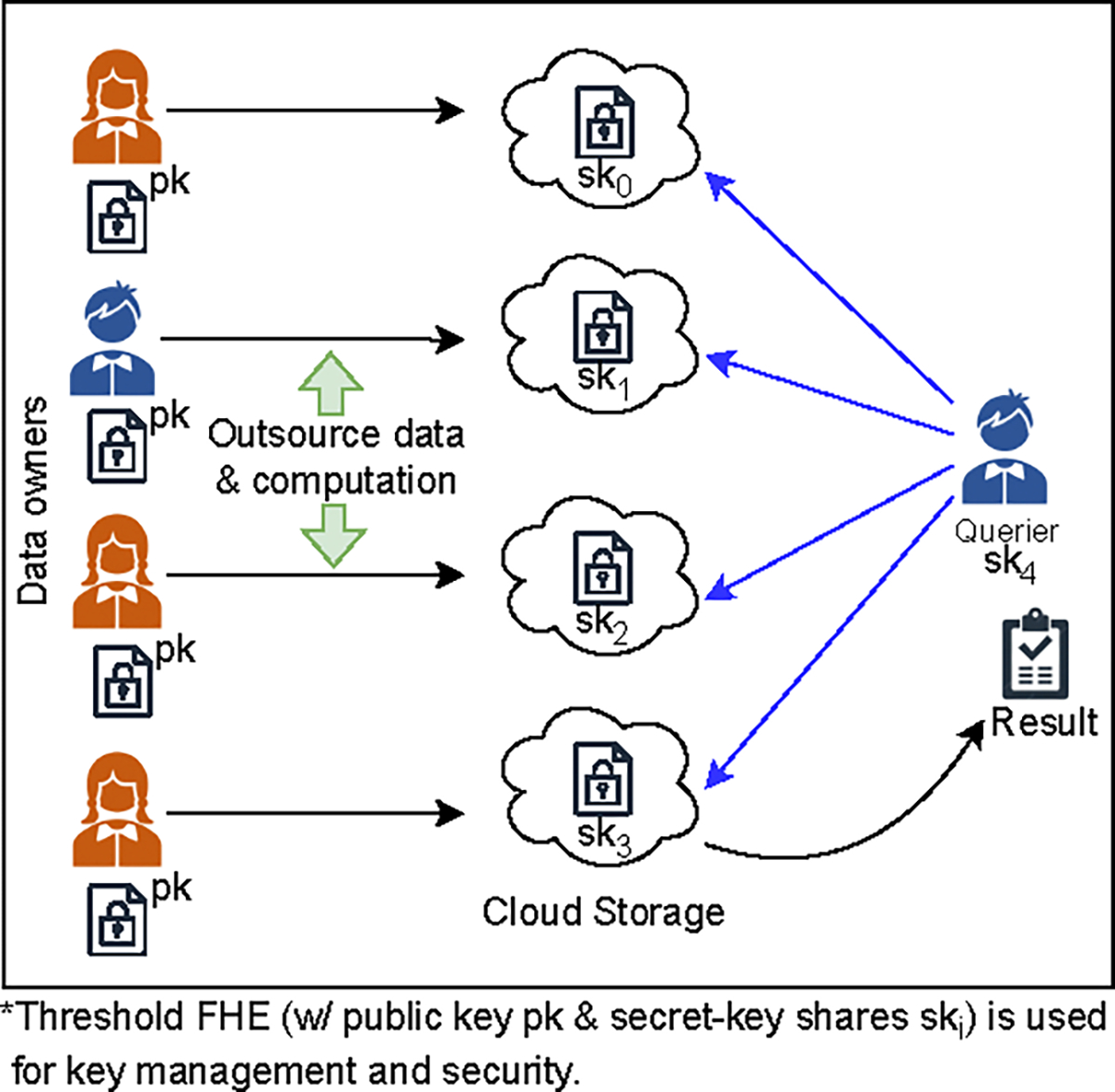
Conceptual illustration of Private Segmented Membership Test (PSMT)

In all the examples above, we face a problem where the number of entities involved in data-sharing applications can be substantial, e.g., thousands of entities when dealing with tax fraud [96]. Furthermore, datasets are updated frequently, and regulations force such datasets containing PII to be stored and operated with strong data security guarantees [37]. These examples all underscore the growing need for privacy-preserving set intersection protocols with a substantial number of dataset holders. There is a need to perform queries by testing the membership of a client’s element within distributed datasets without leaking information about which set the intersection came from. We call this property *provenance privacy*, which refers to the confidentiality of from which party the intersection comes from. These datasets are updated frequently and held by multiple distinct parties, which should be strictly protected to ensure individual privacy. For this purpose, storing and using records in encrypted form can safeguard against data breach attacks or insider threats. This allows data holders (e.g., Amazon AWS or Microsoft Azure) to hold their customers’ (data owners’) data in encrypted form to comply with privacy regulations. These data holders store and operate on the encrypted data provided by their clients or data owners (e.g., financial institutions, hospitals, tax-collecting, and law-enforcement agencies) on their behalf. This scenario is different from those considered by privacy-preserving techniques such as PSI, MPSI, PMT or ORAM (detailed below and in Section 2) in multiple ways. We term this problem in such a scenario with multiple data holders *Private Segmented Membership Test* (PSMT). A conceptual illustration of PSMT is given in Figure 1.

Existing approaches, such as private set intersection (PSI), fall short in numerous ways for those scenarios. PSI enables two parties (referred to as *receiver* and *sender* hereafter by convention [43, 82, 93]) to compute set intersections without revealing any additional information. While existing PSI protocols can address the PSMT, they require dataset holders to access the elements of their sets in plaintext format [23] to enable various optimizations for polynomial interpolation, and they do not scale well with a high number of parties. Thus, for situations with encrypted databases with sensitive information such as medical, financial, or criminal records, PSI is not suitable. Generic multiparty-PSI (MPSI) protocols like [11, 18, 71, 84, 107, 109] are also less suitable for efficiently solving PSMT due to a high number of interactions (in OT-based), privacy concerns against the senders, high aggregation runtime, bandwidth or storage needs. Additionally, Private Membership Test (PMT) protocols [97], optimized for single elements, require unencrypted databases, may produce high false positives due to filters for representing the database [33], and are not designed for multiparty use without compromising the provenance privacy of the senders. ORAM-based (Oblivious RAM) techniques enhance data security by enabling encrypted datasets; however, they cannot handle a high number of servers and multiple clients without a non-collusion assumption and typically require a private state for each client interacting with the ORAM server.

Fully Homomorphic Encryption (FHE) is a widely used building block for constructing PSI protocols due to its single-round communication requirement. However, PSI protocols based on FHE,[21, 23, 38], face several challenges in addressing the PSMT problem, including key management, efficiency, and security issues. Theoretically, given a set held by the sender X and receiver query y, existing PSI protocols homomorphically compute a *sender polynomial*, $f(y)=r\prod_{x\in X}(x-y)$, where r is a random mask. To apply this method for PSMT, each sender would individually calculate their own sender polynomial, after which the *multiplicative* aggregation of these polynomials would correctly be an encryption of zero for an intersection and a random nonzero value otherwise. However, it would require FHE parameters that can tolerate O(log(l)) *additional* multiplicative depth for even a moderately large number of parties l (e.g., l=64). Sender partitioning, although reducing multiplicative depth during polynomial computation, increases depth during result aggregation, particularly with multiple sender polynomials resulting from multiple senders. Moreover, using solely FHE to solve PMST would require non-collusion among all the parties, and a malicious receiver holding FHE secret keys would be able to monitor and decrypt all communications to/from the senders. Threshold-FHE can be used to handle such issues, where secret key shares are distributed to the parties. It facilitates better key management, prevents unauthorized decryption, and avoids a single point of failure if one of the parties acts adversarially.

Our PSMT protocol handles encrypted input for both the receiver’s element and sets held by the sender and does not rely on any preprocessing (besides encryption) of the sets beforehand, completely eliminating the need for the senders to access the sets in plaintext during query computation. Our protocol’s construction is based on threshold-FHE, where the parties use cheap homomorphic additions to aggregate ciphertexts gathered from multiple sites. Using α-out-of-l threshold-FHE, our protocol can handle up to α−1 colluding parties where α<l/2. The security of our protocol is derived from the post-quantum security of FHE [3], and it upholds the provenance privacy of the senders to the receiver. We provide further security countermeasures for adversaries in the IND-CPA^*D*^ model by using existing noise-smudging techniques. We assume a semi-honest model where all parties operate on homomorphically encrypted datasets. In summary, we construct a protocol that can tolerate a large number of senders and efficiently compute on encrypted sender sets that may require frequent set updates.

The contributions of this work are summarized as follows:

- We define the Private Segmented Membership Test (PSMT) problem, which is widely relevant to real-world scenarios. Existing approaches result in various limitations, and we present a novel solution to address them.
- We address the shortcomings of existing PSI and MPSI-based approaches using finite-field FHE for solving PMST, which results from encrypted user data segmented across many senders and high aggregation latency. For the first time, we provide a novel summation-based set intersection protocol with approximate arithmetic threshold-FHE that overcomes these limitations and handles collusion among parties under an honest majority assumption, providing IND-CPA^*D*^ security.
- For the technical challenges in solving the PSMT problem with our novel solution, including plaintext domain size, function approximation accuracy/latency, throughput, and parameterization, we provide concrete parameters and novel strategies to deal with such issues and achieve good performance even for a very large number of senders and set sizes.
- We implement our method and present an experimental evaluation of our solution to show its significant performance advantage in the case of a large number of senders. Our anonymized source code is available for reproducibility and future research at https://anonymous.4open.science/r/psmt-7777. We show up to 2.4× to 5.6× performance improvement over previous works.

## 2 RELATED WORK

### 2.1 Private Set Intersection (PSI)

The first PSI protocol was based on the Diffie-Hellmann (DH) key agreement scheme [78]. This protocol leveraged the commutative properties of the DH function and offered security against the random oracle model. Its low communication cost continues to serve as a foundation for many modern PSIs. Freedman *et al.* [49] introduced PSI protocols based on oblivious polynomial evaluation (OPE) where sets are represented as polynomials. Additionally, PSI protocols have been constructed using Oblivious Pseudo-Random Functions (OPRFs) [48, 63], garbled circuits [90, 110], oblivious transfer (OT), and OT-extension [46, 70, 88, 94, 98]. Recent PSI protocols for unbalanced set sizes use OPE and increasingly utilize FHE with post-quantum security [21, 23, 38, 62, 102].

The two-party Private Set Intersection (PSI) model is extensively studied due to its wide real-world applications. Several variants of this model exist, where either both parties learn the intersection (mutual PSI) [44, 59] or only one of the parties learns the intersection (one-way PSI) [23, 92]. Other variants also allow the computation of different functions on the intersection [36, 44, 59, 60, 111]. Many of these protocols scale to millions of items within seconds and are only slightly slower than the simple but insecure method that exchanges hashed items. Pinkas *et al.* [94] used (1-out-of-n) OT based on [69]. The limitation of their approach is that OT step requires the sender to access elements in the hash table’s bins, and extending it to substantial parties requires multiple Oblivious Pseudo-Random Function (OPRF) evaluations via OT, greatly increasing communication overhead.

CLR17 [23] protocol, and its improved variants [21, 38] are state-of-the-art FHE-based PSI protocols to the best of our knowledge. The basic protocol in [23] has the sender sample a uniformly random non-zero element $r_{i}$ and homomorphically compute the intersection polynomial $z_{i}=r_{i}\prod_{x\in X}\left(c_{i}-x\right)$ using encrypted receiver’s set ($c_{1},c_{2},\ldots ,c_{n}$) and the sender’s unencrypted elements x∈X. $z_{i}$ is returned to the receiver, who concludes that y∈X iff $z_{i}=0$. The receiver only learns the presence of an intersection. The CLR17 protocol applies many optimizations, including receiver and sender side batching using cuckoo hashing and binning, SIMD (Single Instruction Multiple Data) using FHE, and windowing. Later works, [21, 38] used an OPRF preprocessing on the encoded sender set, achieved malicious security, applied the Paterson-Stockmeyer algorithm for evaluating the intersection circuit, and reduced the communication by using extremal postage-stamp bases. These protocols require the sender to access the set in plaintext for the aforementioned optimizations and encodings for creating the polynomial for interpolation. Consequently, the privacy of datasets held by the sender is only protected against the receiver and not against the sender. Fundamentally, the CLR17-based protocols perform PSI by employing zero as a *multiplicative annihilator* in the polynomial $\Pi_{x\in X}(y-x)$. Adapting these methods to the multi-party scenario would drastically increase the multiplicative depth required to obtain the query result and result in scalability issues.

### 2.2 Multi-party PSI (MPSI)

Multi-party PSI (MPSI) extends the two-party PSI problem to scenarios involving more than two parties. Two-party PSI protocols can be extended to multiple parties to handle the MPSI scenario; however, these solutions often lead to privacy and performance issues [108]. Several techniques have been employed to design MPSI, such as circuit-based computations [91], bloom filters [9, 80, 81], OPE [27, 49, 67], and OT and permutation-based hashing [89]. Kolesnikov *et al.* [71] used a technique based on oblivious evaluation of a programmable pseudorandom function (OPPRF) to implement a time-efficient MPSI protocol for large amounts of items. However, their time complexity scales quadratically w.r.t the number of parties in the protocol. Chandran *et al.* [18] improves upon [71] in terms of communication and extends it to circuit-based PSI and quorum-PSI. Notably, these protocols provide intersection results to all or some parties based on the intersection outcome and do not support a substantial number of parties.

Badrinarayanan *et al.* [7] employ threshold FHE to construct threshold MPSIs with sublinear communication complexities with thresholds proportional to the number of elements in datasets. They use a similar polynomial encoding of each set element as in [23]. Bay *et al.* [8] provide two MPSI protocols based on bloom filters and threshold homomorphic public-key techniques. Their protocol performs better than previous state-of-the-art [71] in terms of run time, given that the sets are small and a large number of senders exist. Nevo *et al.* [84] construct concretely efficient malicious MPSI protocols based on the recently introduced primitives such as OPPRF and oblivious key-value store (OKVS).

### 2.3 Other Similar Methods

#### Private Membership Test (PMT).

PMT, or Private Set Inclusion, is a similar problem to PSI, in which a receiver learns if their single element is included in a sender’s database without revealing anything to the sender. To solve PMT, many works apply Private Information Retrieval (PIR) based protocols that allow a user to retrieve an item from a database without the database owner learning anything about the item [35, 51, 73, 83]. PSMT closely matches the PIR; however, the sender’s database is public in PIR. PMT has been extensively studied, particularly for two-party PSI in malware detection [97]. While hashing seems a naive solution for low-latency multi-party PMT, it becomes insecure with low-entropy input domains, and even high-entropy input domains, it may leak repeated elements upon consecutive executions. One can solve PSMT using individual PMT protocols with all senders via 1-out-of-n OT-based PMTs, followed by a secure XOR computation by the client. However, this approach has several drawbacks. Firstly, it necessitates sender access to plaintext sets for OT, losing privacy. Secondly, it requires the client to run n PMT protocols with n senders, adding extra communication and computation. While OT extension-based protocols can reduce communication, they also demand access to plaintext sets, and any updates in databases result in significant performance and communication penalties.

Some works have applied the PIR protocols to the PMT problem [33, 97] based on homomorphic encryption and bloom filters, but they induce significantly high false positives. Kulshrestha *et al.* [72], and Wang *et al.* [106] have constructed PMT protocols for identifying harmful media content and to detect password reuse across multiple websites, respectively. Tamrakar *et al.* [103] propose a carousel method for PMT for solving malware detection based on Trusted Execution Environments (TEEs). However, TEEs suffer from side-channel attacks and hardware-based attacks [20, 54, 77, 105], which have decreased their confidence for use recently.

#### Oblivious RAM (ORAM).

ORAM allows a client to outsource storage of data to a server, and enable read and write operations to that data without revealing anything to the server about the data [53]. Many state-of-the-art ORAM frameworks [57, 58] require only constant client bandwidth blowup and low client storage but rely on weaker non-cryptographic security assumptions. ORAM-based techniques can be employed to solve PSMT but only partially. Namely, it is primarily designed for a single private database that can only be accessed by a single client, and using multiple servers requires a strong non-collusion assumption between them [57]. Distributed ORAM (DORAM) is a variant that handles multiple non-colluding servers and can be used for PSMT, but data is duplicated across the servers in DORAM. Furthermore, DORAM has higher bandwidth requirements and induces significant overheads while scaling for a higher number of senders. DUORAM [104] is one of the state-of-the-art DORAM models, however, it provides instantiations for up to only 3-party computation, which is far less than the scale involved in our scenario. Although the database on the server is encrypted in ORAM (similar to PSMT), ORAM requires the client to have a private state, due to which multiple clients cannot interact with the ORAM server directly, and more importantly, any querier who doesn’t have access to this state cannot interact with the server.

In summary, PSMT can be addressed using general PMT-solving methods based on PIR, OT, ORAM, or TEEs, but they only offer partial solutions. The considerable overhead for a large number of senders, a lack of privacy for datasets held by senders, either from the client or the sender(s), along with high latency and low throughput typically associated with PMT protocols [103], render them impractical for efficient PSMT solutions. We compare representative works to our work in Table 1.

## 3 PRELIMINARIES & DEFINITIONS

In this section, we summarize some of the important notations for FHE and PSMT. We provide a complete list of notations in Table 2.

### 3.1 Fully Homomorphic Encryption

Fully Homomorphic Encryption (FHE) is a cryptographic primitive that allows computation on encrypted data with post-quantum security. Noise associated with an FHE ciphertext grows corresponding to each homomorphic operation, i.e., additively with additions and multiplicatively with multiplications. The most prominent FHE schemes are BGV [17], B/FV [15, 47], CKKS [29], and TFHE [34]. In practice, FHE schemes are often implemented as Somewhat Homomorphic Encryption (SHE) schemes where the user(s) provide the multiplicative depth required by the computation at the setup phase. In this work, we use the CKKS scheme, which uses a fixed-point complex number encoding to enable homomorphic computations on real numbers. Similar to B/FV and BGV, CKKS has operands in $ℛ=Z[X]/\left\langle \Phi_{M}(X)\right\rangle$, where $\Phi_{M}(X)$ is the cyclotomic polynomial $\left(x^{N}+1\right)$ of order M=2N (cyclotomic index) and degree N∈Z which is the ring dimension. CKKS parameters include the ring dimension, ciphertext modulus, and standard deviation of the error. We employ the CKKS parameters to maintain 128-bit security in both classical and quantum contexts [4, 74, 79].

#### SIMD:

In FHE, we can consider the factorization of $\left(x^{N}+1\right)$ modulo p where p is a prime. We can then write the message space as a direct product of small fields, encrypt a vector of elements of these fields, and operate in parallel on the entries of these vectors, thus obtaining single instruction, multiple data (SIMD) capabilities [101].

In general, we have $\left(x^{N}+1\right)=f_{1}(X)\cdots .f_{\eta }(X)$ (mod p) with all $f_{i}$’s having the same degree d such that N=η⋅d and message space is $Z_{p}[X]/\left\langle x^{N}+1\right\rangle =\prod_{i=1}^{\eta }\left(Z_{p}[X]/\left\langle f_{i}(X)\right\rangle \right)={\left(F_{p^{d}}\right)}^{\eta }$. The plaintext space is isomorphic to η copies of the finite field with $p^{d}$ elements, and instead of encrypting one single high-degree polynomial, we can encrypt a vector of η elements of $F_{p^{d}}$. Therefore, a single homomorphic operation can handle η messages, each stored in a ciphertext slot, with the total slots and batch size equals η.

### 3.2 Threshold FHE

For the threshold functionality in our protocol, we utilize α-out-of-l (leveled) threshold-FHE (thresFHE) where l is the number of parties and α is the minimum number of partial decryptions needed to complete the decryption [12]. A thresFHE scheme consists of a tuple of probabilistic polynomial time (PPT) algorithms (*ThresFHE.Enc*, *ThresFHE.Eval*, *ThresFHE.PartialDec*), and two l-party protocols (*ThresFHE.KeyGen*, *ThresFHE.Combine*) with the following functionalities:

- $ThresFHE.KeyGen(1^{\lambda },1^{D},params)\to (pk,evk,{\left\{sk_{i}\right\}}_{i\in [n]})$ : Given a security parameter λ and a depth D, each party $P_{i}$ outputs a common public key pk for encryption, a common evaluation key evk, and a secret key share $sk_{i}$ of the implicitly defined secret key sk under some public parameter *params*.
- ThresFHE.Enc(pk,m)→c: Given a public key pk, a message m, the encryption algorithm uses error distributions $\chi_{enc}$ and $\chi_{err}$ to sample $u\leftarrow \chi_{enc}$ and $e_{0},\;e_{1}\leftarrow \chi_{err}$ and outputs $c\leftarrow u\cdot pk+\left(m+e_{0},e_{1}\right)$ mod q such that, $c=\left(c_{0},c_{1}\right)$ where the ciphertext space is defined as ${ℛ}_{q}^{2}=(ℛ/\langle q\rangle {)}^{2}$.
- $hresFHE.Eval(evk,f,{\left\{ck_{i}\right\}}_{i\in [v]})\to c^{*}$: Given an evaluation key evk, a v-input function, f that can be evaluated using at most depth D and ciphertexts $c_{i}$, the evaluation algorithm outputs a new ciphertext $c^{*}$ that is an encryption of $f\left(m_{1},\ldots ,m_{v}\right)$. where $c_{i}\leftarrow ThresFHE.Enc\left(pk,m_{i}\right)$.
- $ThresFHE.PartialDec\left(c,sk_{i},\chi_{smg}\left(B_{smg}\right)\right)$: Given a ciphertext $c=(c_{0},c_{1}$), a secret key share $sk_{i}$ and a smudging error distribution $\chi_{smg}\left(B_{smg}\right)$ with a bound $B_{smg}$, the partial decryption algorithm samples a smudging error $e_{i}^{\mathrm{smg}}\leftarrow \chi_{\mathrm{smg}}\left(B_{smg}\right)$, and computes ${pdec}_{i}\leftarrow c_{1}\cdot s_{i}+e_{i}^{smg}$.
- $ThresFHE.Combine(pk,{\left\{pdec_{i}\right\}}_{i\in [I]})\to m$ or ⊥: Given a public key pk, a set of partial decryptions ${\left\{pdec_{i}\right\}}_{i\in [I]}$ for an index set I⊆[n] the combine algorithm computes $c_{0}+\sum_{i=0}^{I}p_{i}$ mod q and outputs m if |I|≥α otherwise ⊥.

The key generation phase in thresFHE can be accomplished using a trusted setup procedure which can be run either via trusted hardware or secure multiparty computation to broadcast partial secret key to α key-holders. Existing works [6, 65] have shown that the latter method of key generation can be completed in a two-round, l-party protocol to compute a common public key, a common public evaluation key and a private share of the implicitly defined secret key. Similarly, thresFHE final decryption is a one-round α party protocol. As in standard homomorphic encryption schemes, we require that a thresFHE scheme satisfies compactness, correctness, and security [7].

### 3.3 Private Segmented Membership Test (PSMT)

#### Problem Definition:

**Figure 2:**
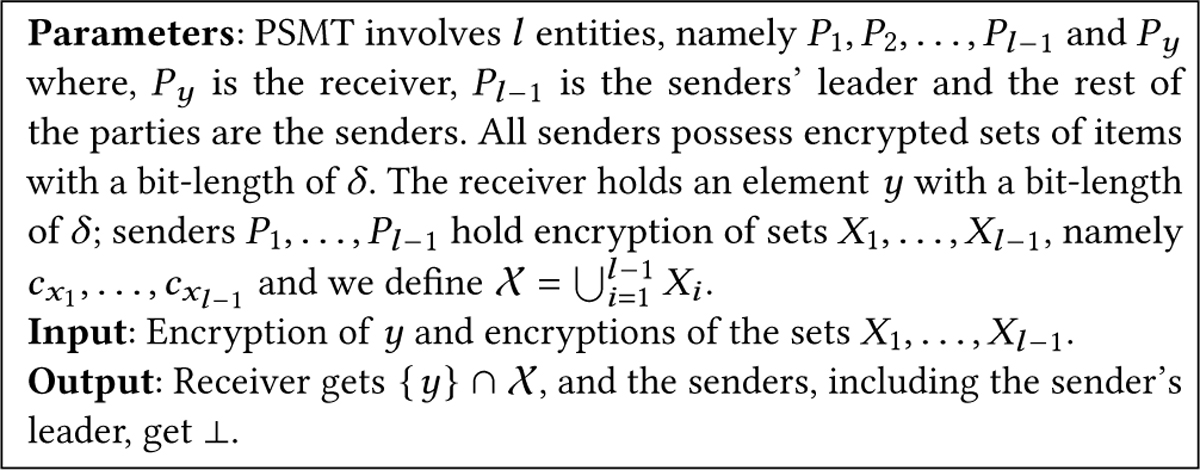
Ideal functionality of PSMT

The PSMT problem is described in Figure 2. For a party $P_{y}$, with a data element y, and l−1 parties $P_{1},P_{2},\ldots ,P_{l-1}$, each with a set $X_{i}$ such that $𝒳=\sum_{i=1}^{l-1}X_{i}$, a PSMT allows the party $P_{y}$ to learn {y}∩𝒳, without leaking any elements not in any $X_{i}$ or which party holds an element in the intersection. The sets held by different senders in the PSMT are mutually exclusive or disjoint. The parties involved are referred to as the *sender* and the *receiver*. For strong protection of user data, each sender in PSMT, $P_{i}$ does not have plaintext access to $X_{i}$, i.e., each sender only has encryptions of $x\in X_{i}$. PSMT outputs $\{y\}\cap {⋃}_{i=1}^{l-1}X_{i}$ to the receiver and nothing to the senders without leaking any other information about the receiver’s element and sets held by the senders.

#### Threat Model:

PSMT protects the outsourced data owned by the data owners from the semi-honest senders, and our threat model is similar to that in previous works [10, 95]. The provenance privacy of the senders is preserved in PSMT. This ensures that in the event where $y\in X_{i}$, the set $X_{i}$ is hidden from the receiver, who only learns that y is in some sender’s set and not any particular set. We do not consider membership inference attacks with repeated adaptive queries. Such attacks can be mitigated by rate limiting or OPRFs [21] at the cost of additional overhead from preprocessing. Since parties are semi-honest, they are guaranteed to use the actual inputs; therefore, there is no integrity tampering [56]. We also do not consider integrity attacks where an adversary may homomorphically modify the sender’s set. Securely storing hashes of ciphertexts (e.g., in an isolated storage) can prevent this.

#### Adversary Model:

We assume a semi-honest setting, where parties (both senders and receiver) are assumed to be honest but curious. For the threshold functionality, we employ α-out-of-l thresFHE that can handle α−1 colluding participants where α<l/2 such that the majority of the participants are honest. Adversaries will try to learn any of the receiver query y (if the receiver is not compromised) and the sender’s set elements $x\in X_{i}$. Adversaries may eavesdrop on messages or compromise up to α−1 parties.

We note that in the rest of this paper, senders’ sets refer to the encrypted sets of data owners that are held by the senders.

## 4 PSMT PROTOCOL

### 4.1 Basic Protocol without Approximation

Protocols using FHE to implement PSI [21, 23, 38] use zero as a multiplicative annihilator. Multiplications are not scalable, so we propose the novel idea of using additive aggregation.

**Figure 3:**
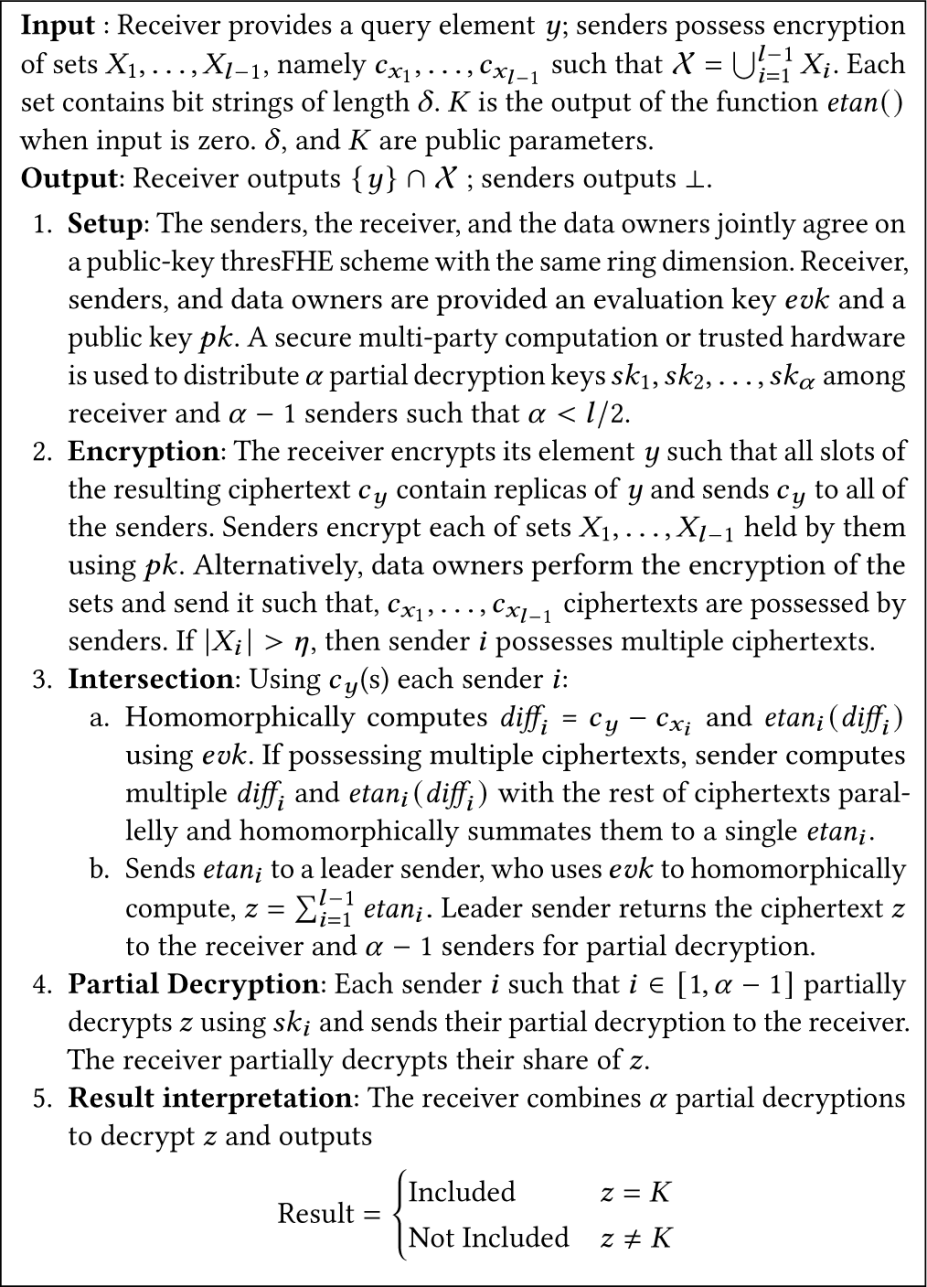
Basic PSMT protocol

We consider set elements to be members of Z (e.g., hashed elements). We describe our basic protocol in Figure 3 as a strawman protocol. The receiver has access to a query element y, and s/he creates replicas of y such that the number of replicas equals η (batch-size) and encrypts them to obtain $c_{y}$ such that all slots of $c_{y}$ contain y. The receiver then sends $c_{y}$ to l senders. Senders either encrypt their sets themselves or receive the encryptions from data owners through a secure communication channel. Thus, each sender i∈[1,l−1] has access to an encryption of a set $X_{i},\;c_{x_{i}}$. Each slot of $c_{x_{i}}$ contains different elements of $X_{i}$. Due to batching, each sender possesses only a single ciphertext. In case $\left|X_{i}\right|>\eta$, the sender possesses multiple $c_{x_{i}}$. Each sender i then computes a homomorphic difference of $c_{y}$, and its ciphertext(s) $c_{x_{i}}$ in a SIMD fashion to obtain ${diff}_{i}$. Each sender i then computes a piecewise function ${etan}_{i}()$, defined below.

$${etan}_{i}\left({\mathrm{diff}}_{i}\right)=\left\{\begin{array}{ll} K, & \;\text{if}\;{\mathrm{diff}\;}_{i}=0\\ 0, & \;\text{if}\;{diff}_{i}\neq 0 \end{array}\right.$$

${etan}_{i}()$ maps the input to K if the input is zero and zero otherwise. If equipped with multiple ${diff}_{i}$, senders compute separate ${etan}_{i}()$ for every ${diff}_{i}$ in parallel since they are independent. The sender then simply sums the ${etan}_{i}$ into a single ciphertext. The results obtained by the senders can be additively aggregated by computing $\sum_{i=1}^{l-1}{etan}_{i}$, whose value is non-zero for an intersection and zero for a non-intersection. If the senders’ sets are disjoint, the summation value will be K for an intersection; otherwise, it will be some multiple of K. We note that in practical deployments, it is most probable that some leader sender will take on the additional burden of aggregation or that different senders will take turns doing this. In our protocol, a leader sender aggregates the results.

This basic protocol can be implemented with any FHE scheme, and the aggregation would be composed of efficient homomorphic additions only. Unfortunately, this version would suffer from high multiplicative depth when computing the condition “if ${diff}_{i}=0$.” This leads to the need to approximate the function ${etan}_{i}()$.

### 4.2 Novel Value Annihilating Function (VAF)

One naïve thought is to approximate the function ${etan}_{i}()$ with a polynomial, but doing so would require polynomials with high degrees. We begin by considering the computation a single sender must perform. Suppose there exists a small negligible value ϵ and a sender has access to a set $X_{i}$ with size n such that $x_{j}\in X_{i}$ for j∈[n] and is given an FHE ciphertext $c_{y}$ encrypting a receiver’s input y∈Y.

Consider the function $f\left(y,X_{i}\right)=\sum_{j=0}^{n-1}g\left({diff}_{j}\right)$, where ${diff}_{j}=\left(y-x_{j}\right)$ and $g\left({diff}_{j}\right)=\frac{1}{{diff}_{j}+\epsilon }$. Then, $f\left(y,X_{i}\right)$ can be thought to exhibit the following behavior similar to that of ${etan}_{i}$:

$$f\left(y,X_{i}\right)=\left\{\begin{array}{ll} 1/\epsilon +\sum_{j=1}^{n-1}g\left({\mathrm{diff}}_{j}\right), & \;\text{if}\;{\mathrm{diff}}_{0}=0\\ \sum_{j=0}^{n-1}g\left({\mathrm{diff}}_{j}\right)\ll K, & \;\text{if}\;{\mathrm{diff}}_{j}\neq 0 \end{array}\right.$$

Here, if ${diff}_{0}=0$ (i.e., $y\in X_{i}$) and $\epsilon \to 0,\;f\left(y,X_{i}\right)$ increases without bound and its limit approaches infinity. This misuses the notion of infinity in terms of computation, as infinity is not a numerical quantity. Nevertheless, it leads us to some intuition on how to construct a protocol that is amenable to PSMT: we should have a function that each sender computes that outputs very large values (close to K) to annihilate the summation on an intersection but outputs a negligible summation value compared to K on a non-intersection. Then, additively summing these results from each sender would yield a result indicating if the receiver’s element y is in $\overset{l-1}{\underset{i=1}{⋃}}X_{i}$, i.e., the large value indicates intersections and small negligible values indicate non-intersections.

To realize this idea, we consider a candidate function $D_{K}^{\mathrm{Exact}}(x):R\to R$ that exhibits the useful properties described above without the problematic behavior of a potential division by zero:

$D_{K}^{Exact}(x)=\left\{\begin{array}{ll} K, & \text{if}\;x=0\\ 0, & \text{if}\;x\neq 0 \end{array}\right.$. This function $D_{K}^{Exact}()$ easily satisfies these constraints and has the exact same behavior as ${etan}_{i}()$. We call such functions *Value Annihilating Functions* (VAF). A VAF maps zero to K and all others to zero. Computing VAFs homomorphically is challenging because *if* conditions cannot be evaluated efficiently in FHE. We thus consider another function approximating $D_{K}^{\mathrm{Exact}}(x)$. Instantiating a VAF on finite fields with exact schemes would require a very large plaintext domain for representing inputs, and the approximation procedure would be very inefficient. Hence, for such a function, approximate-arithmetic FHE is preferable.

It is challenging to find a function that accurately approximates such kind of behavior. The functions approximating such kind of behavior require computing the inverse or the division operation, and computing such an operation homomorphically is fundamentally challenging. Similarly, trying to control the maxima of such functions while approximation results in very bad accuracies. We explored many candidates for VAFs, such as rational functions including $f_{K,S}(x)=\frac{K}{{\left(1+S\cdot x\right)}^{4}}$, sigmoid, hyperbolic tangent, piecewise linear, sign function, among others. The rational functions require computing a division for VAF, which is inefficient for FHE. Sigmoid, hyperbolic tangent, piecewise linear, and sign functions either did not exhibit the kind of properties that we needed or resulted in a very high accuracy loss while approximating in FHE. With all the considerations, we discovered the following function exhibits satisfactory behavior for our purpose with minimal accuracy loss:

$$D_{K,S}^{Approx}(x)=K\cdot \left(1-{tanh}^{2}(S\cdot (x))\right)$$

This function is shown in Figure 5, approximating a VAF. Since piece-wise functions cannot be evaluated efficiently in FHE due to branching, we designed a function that gets larger quickly as the input gets closer to zero. The derivative of the hyperbolic tangent $\left(1-{tanh}^{2}(x)\right)$ has maxima as one for zero inputs and approaches zero for inputs in the range (−∞,−3)∪(3,∞). We set K acts as the maxima and use S to increase the input range for zero (i.e., width of the function’s hump near the zero) such that the function outputs zero for inputs in range (−∞,−1)∪(1,∞). Using this function for approximation, senders acquire a summation value that is equal to K when an intersection is detected; otherwise, they obtain a summation value that is close to zero.

When computing functions such as $D_{K,S}^{Approx}(x)$ in approximate homomorphic encryption, only additions and multiplications are available. However, such approximations are not zero-valued for all nonzero arguments or even all arguments outside some interval centered at zero. In the following sections, we describe how we compute a polynomial approximation $D_{K,S}^{Approx}(x)$ whose values on R except for a small interval near zero can be bounded by some small number κ. Then, we can set parameters K,S,κ,v such that (l−1)⋅κ<τ, where τ=v⋅K.v∈(0,1] is a threshold proportion used to account for the case where error from approximate-arithmetic FHE may cause a sum of l outputs of $D_{K,S}^{Approx}(x)$ on nonzero arguments to be greater than (l−1)⋅κ.

### 4.3 Polynomial Approximation of VAF

The approximation of complex non-polynomial functions is a well-studied topic. Existing works such as [30, 31] use polynomial composition with iterative algorithms to approximate functions like min/max and comparison using FHE. Other works have used techniques like Taylor series [13, 66], minimax approximation [28, 100], look-up tables [40] and conversion between FHE schemes [10, 34].

A major challenge for approximation techniques is finding polynomial approximations that work on large domains. Existing approaches for approximation [13, 29, 64, 66] struggle for large domain intervals, inducing very high approximation errors. Even domains of thousands can be challenging [32, 64]. This would require homomorphically evaluating polynomials of an extremely large degree. For instance, simply using Chebyshev-based approximation for approximating non-linear functions for domains such as [−1000, 1000], requires almost 20.5 minutes of computation time and an additional 12 multiplicative depth for acceptable accuracy. Hence, the error induced by the approximation and the computational cost of approximation are the major factors [32] to be considered during the approximation. For our application, we are primarily concerned with the computational cost of the evaluation and require low precision that will enable us to distinguish values converging to K (for intersection) and 0 (for non-intersection).

#### Domain Extension Polynomials (DEPs).

DEPs enable the effective shrinking of a large domain interval $\left[-L^{n}R,L^{n}R\right]$ to a smaller subinterval [−R,R] such that the property of the VAFs around zero in the smaller domain is preserved. To compress inputs from an interval $\left[-L^{n}R,L^{n}R\right]$ to an interval [−R,R], we can iteratively apply a DEP with O(n) operations and 2n additional depth. Using this method we can convert inputs $z\in \left[-L^{n}R,L^{n}R\right]$ into values D(z) such that z∈[−R,R]⟹D(z)≈z and z∉[−R,R]⟹D(z)≈sign(z). Specifically, we utilize Algorithm 1 from [32]. To handle inputs in [−R,R] using the DEP $B(z)=z-\frac{4}{27}z^{3}$ at an iteration n of Algorithm 1, we divide inputs to B(z) by $L^{n}R$, and scale its ouputs by $L^{n}R$. This converts B(z) from a DEP on [−1,1] to a DEP on [−R,R]. If the accuracy of B(z) is not good enough, then squaring its outputs can make B(0) larger and B(z) for z≠0 smaller, at the cost of additional depth and runtime. DEPs enable the approximation of values within a large domain interval, allowing the protocol to handle potentially millions of inputs efficiently.

#### Chebyshev Approximation.

Chebyshev polynomial approximation are minimax-based polynomial approximation method that achieves the smallest possible polynomial degree with minimal approximation errors [32, 64]. Approximating functions using standard approximation techniques like Taylor series can induce the Runge phenomenon [14] that causes an approximation to yield poor accuracy at the edges of the interval. Chebyshev polynomials reduce this phenomenon and provide an approximation that is close to the best polynomial approximation to a continuous function.

We apply DEPs and Chebyshev approximation to approximate a $D_{K,S}^{\mathrm{Approx}}(x)$ in a much smaller interval [−R,R] while preserving the original larger domain $\left[-L^{n}R,L^{n}R\right.]$ with low FHE multiplicative depth. This leads our protocol to approximate ${etan}_{i}()$ with low approximation errors and computational cost for a large domain.

### 4.4 Security

Using our basic protocol in Figure 3, the receiver can learn the aggregation value $\sum_{i=1}^{l-1}{etan}_{i}$ to infer information about the difference between their query and the sender’s value to get information about the non-intersection values. To fix this issue, we require the senders to obscure the aggregation value by adding a small random masking term r∈[−κ,κ], where κ is a public bound for the approximation value of VAF on a non-intersection.

Our protocol incorporates a threshold version of the CKKS scheme to withstand collusion among up to α parties where α<l/2. This ensures that even if the receiver acts as an adversary, they cannot derive any additional information from the communication channels, as decryption is unachievable with only their single partial key. Consequently, the receiver would need to collude with α−1 parties to gain any extra information. Semantic security of our PSMT protocol follows directly from the security of the underlying threshold variant of the FHE scheme CKKS [29]. Recent works by Cheon *et al.* [25] and Li and Micciancio [75] have shown that IND-CPA security is not sufficient for both exact (BGV, BFV, and TFHE) and approximate (CKKS) FHE schemes. In particular, [75] presented a passive attack against CKKS and showed that CKKS is not secure if access to a decryption oracle is provided. They introduced a new notion of privacy called IND-CPA^*D*^ that provides access to encryption, evaluation, and decryption oracles. Moreover, the access to decryption oracle requirement is also present in thresFHE schemes for accessing the partial decryptions, and thus the threshold variants of all FHE schemes, including CKKS in our basic protocol, are exposed to IND-CPA^*D*^ attackers [19, 68].

#### Handling IND-CPA^*D*^ capable adversaries.

In CKKS, the noise component is a part of the message, and this results in the linearity of the decryption function to the secret key revealing the decryption noise, and making it vulnerable to IND-CPA^*D*^ attackers [26]. To mitigate this kind of attack, existing works [19, 26, 42, 75], have proposed various countermeasures such as bootstrapping to reset the noise variance to a preset value, using the rounding procedure or attaching a proper noise at the end of the decryption process called smudging noise to avoid the linearity on the secret key. We employ the noise smudging (noise flooding) method to transform the thresFHE CKKS scheme achieving the weak IND-CPA security definition into one which is IND-CPA^*D*^ secure [76].

Before adding the noise, proper estimation needs to be done for noise addition using either static or dynamic noise estimation [5, 76]. In our application, we use static noise estimation since it can be performed offline to compute the noise using publicly available bounds on the inputs and the function to be evaluated. Senders estimate the noise using a fresh secret key-public key pair and select messages reflecting actual data for the homomorphic computation. The additional noise is drawn from $D_{R,\sigma }$ over the polynomial ring, represented in its coefficient form where σ is a standard deviation set by a security level and noise estimate. Thus, to achieve s>0 bits of statistical security, one can set $\sigma =\sqrt{24a\cdot N}\cdot 2^{s/2}$ (see Corollary 2 in [76]), where a is the number of adversarial queries the application is expecting, and N is the ring dimension used for FHE. We add smudging noise using this distribution to ensure IND-CPA^*D*^ security for our underlying thresFHE CKKS scheme.

### 4.5 Choosing Appropriate Parameters

Over the course of our experiments, we encountered several challenges in determining suitable parameters for the DEP and Chebyshev approximation and tailoring them to the specific sender set sizes. To set up the DEP parameters for reducing the Chebyshev approximation degree, one needs to be careful while choosing L,R, and n with c to minimize FHE depth and approximation errors for a given |𝒳|. We provide further details regarding parameterization and list our findings in Appendix A and Table 5.

We note that the values described in this section and in Table 5 are useful for the case where $\overset{l-1}{\underset{i=1}{⋂}}X_{i}=Ø$, i.e., each sender’s set has no element in common with other sender sets. In case senders’ sets are not disjoint, we can allow up to Ψ of l senders to have a duplicate element, so long as values up to Ψ⋅K can be represented correctly in CKKS with the parameterization being used. To ensure that the summation does not overflow, we can set a smaller K and heuristically choose smaller values for parameters j,k,ρ, and τ. Note that in case, we allow duplicate items, our protocol will require higher precision bits to accommodate for the precision loss occurring during smudging noise addition for security.

#### Smudging Noise.

In our protocol, we require the senders to add a smudging noise to their partial decryptions from $\sigma =\sqrt{24a\cdot N}\cdot 2^{s/2}$ that has a larger variance than the standard noise distribution of basic thresFHE CKKS. We set $a=2^{10}$ and s=36, limiting the adversary’s success rate to 2^−36^ (about 1 in 68 billion) for 2^10^ adversarial queries for a single ciphertext. Li and Micciancio offer noise-estimation parameters for $a<2^{15}$ only (see Table 1 in [76]) and having higher values ofa without adding larger smudging noise or significant precision loss is a problem orthogonal to ours and a topic under research [19, 39, 76]. Hence, we assume that distributed thresFHE queries for identical or related ciphertexts are limited to fewer than 2^10^ in our application since all parties will need to be involved in every thresFHE decryption. One solution to mitigate such an issue would be refreshing the noise estimate after every 2^10^ queries which would add minimal overhead to the latency as it can be done offline. Similarly, another mitigation as suggested by [76], would be updating the secret key shares occasionally, however, this method will require extra rounds of communication.

After running the noise estimation, we estimated roughly 34 noise bits for smudging. This smudging noise ensures the IND-CPA^*D*^ security; however, it introduces noise with a high variance to each partial decryption. Moreover, the noise amplifies when aggregated across numerous parties, leading to a substantial loss in precision due to which non-intersection summation $z=\sum_{i=1}^{l-1}{etan}_{i}$ can overflow beyond negligible values. However, this is not an issue in our protocol since we can simply choose a higher τ value to accommodate for the precision loss. Moreover, the noise smudging is performed independently by the senders, which incurs very low latency and, thus, does not significantly affect the protocol’s latency.

### 4.6 Asymptotic Complexity Analysis

**Figure 4:**
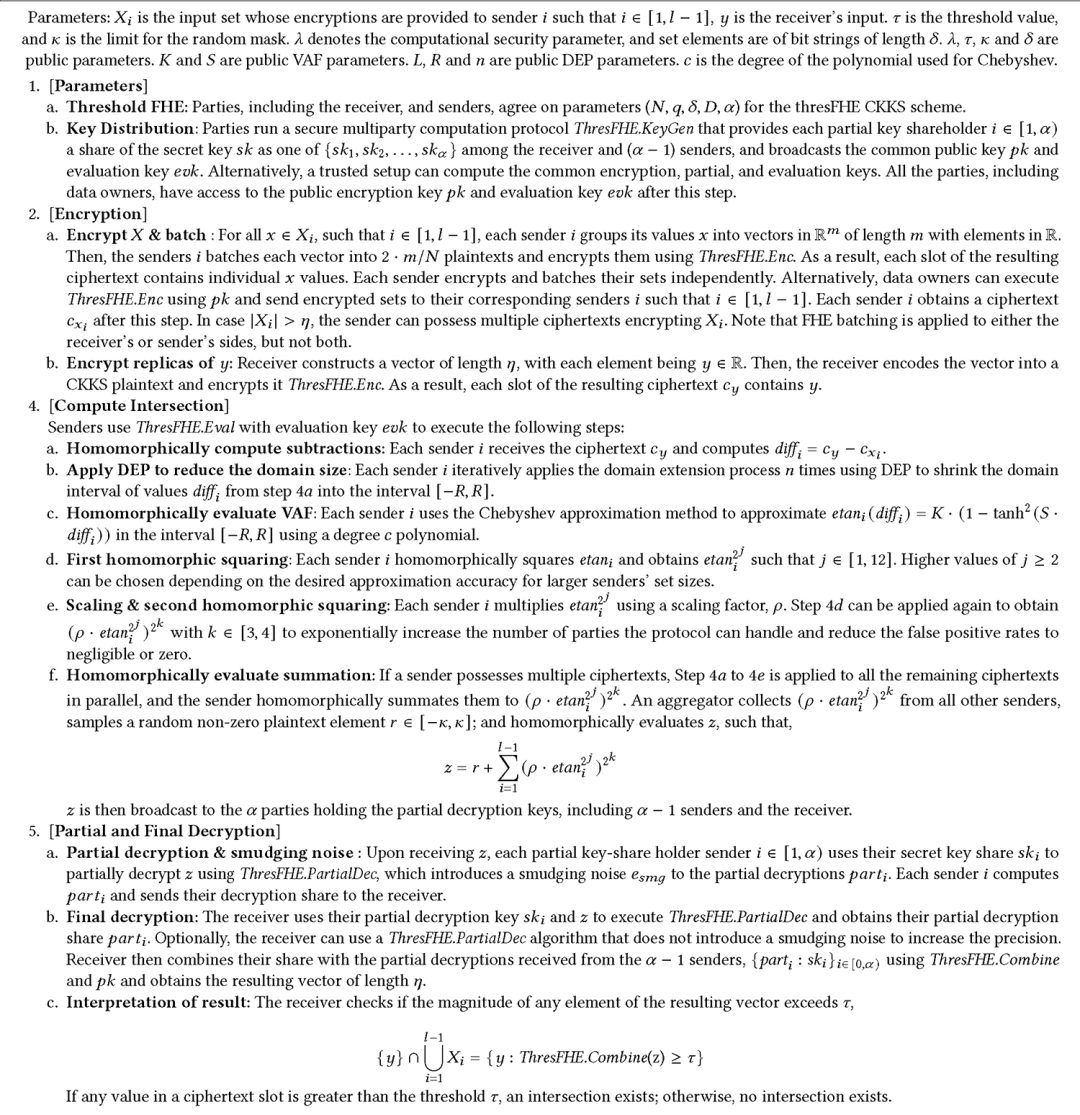
Full PSMT protocol

**Figure 5:**
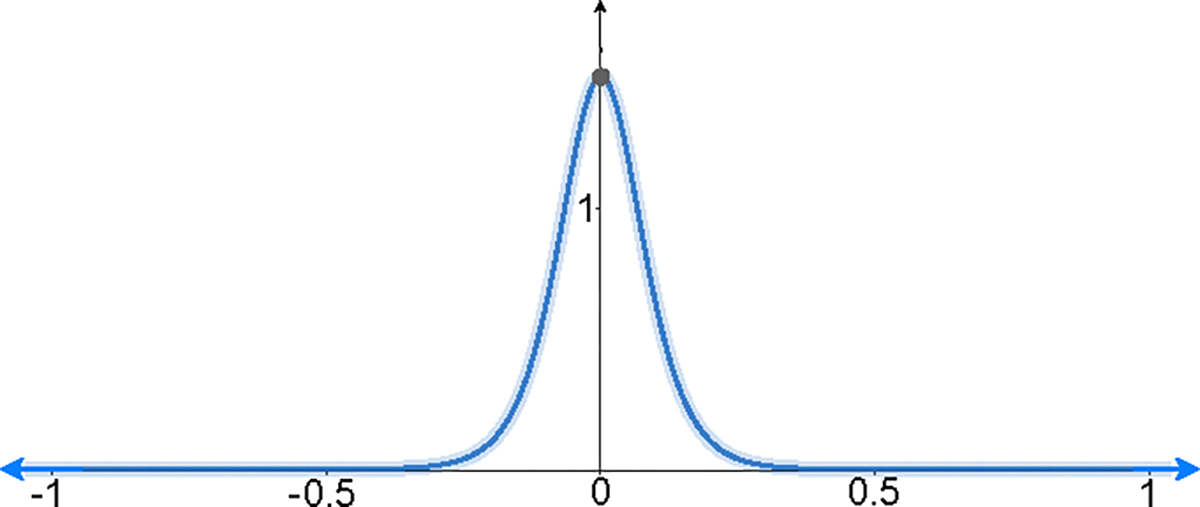
**Graph of the VAF** $K\cdot \left(1-\tanh^{2}\left(S\cdot x\right)\right):K=1.5,\;S=10.$

Each sender must compute the procedures described in Figure 4. This requires 2n+O(log(c))+j+k homomorphic multiplications. These procedures require a multiplicative depth of 2n for the application of the DEP, approximately log(c)+1 for Chebyshev approximation, and j+k for repeated squaring. The precise amount of depth recommended for a polynomial approximation of degree c is chosen heuristically^1^. CKKS operations besides multiplication, e.g., addition and scaling, contribute relatively small amounts of overhead and noise. The additive aggregation of each sender’s result is relatively inexpensive; most notably, it does not increase multiplicative depth *even with an increasing number of senders*.

Our protocol requires sending one ciphertext from the receiver to the l−1 senders and receiving α−1 ciphertexts from the senders by the receiver. Thus, the communication between the receiver and senders is bounded above by l+α−2 ciphertexts. The inter-sender communication is bounded by (l−1)·α ciphertexts which includes the final result ciphertext sent to the receiver by the aggregator. The number of communication rounds between the receiver and senders is four in our protocol, assuming a trusted setup provides all the parties their respective keys. In case a separate secure multiparty protocol is run for the key generation during the setup phase, it will add two more rounds to the overall communication.

### 4.7 Discussion

#### 4.7.1 Set Updates.

As we do not require any preprocessing of senders’ sets, updates to the encrypted senders’ sets are trivial in our protocol. Most computations in our protocol take place during the online phase of the protocol. This allows the protocol to easily add or delete any element in senders’ sets, as no pre-processing is necessary during the offline phase. The data owners can simply update their sets, encrypt them, and send the corresponding ciphertext(s). Alternatively, data owners can only send the latest set of elements separately, which will save communication costs. Senders can then use all received ciphertexts to compute the PSMT for new queries. This will allow the parties to compute the intersection of their private sets on a regular basis with sets that are often updated.

#### 4.7.2 Multiple Receivers.

In case we have multiple receivers, each receiver must participate in the setup phase of key generation to obtain a partial decryption key. The threshold value α can be adjusted to accommodate the increased number of receivers. For r number of receivers, the threshold can be adjusted to (α−r) which will ensure that only the querying receiver needs to supply partial decryption to complete the decryption process. However, with multiple receivers, it’s crucial they do not collude, as each holds a partial decryption key, and collusion can lead to fewer than (α−r) senders being required for decryption, as malicious receivers could get access to the final result by monitoring the communications channels. In scenarios where a new receiver wants to perform the query, we can update the secret key shares in thresFHE by updating the original polynomial used for setting up secret sharing.

#### 4.7.3 Attacks on CKKS in OpenFHE.

For our experiments, we utilize an open-source FHE library called OpenFHE [2]. OpenFHE uses non-worst-case noise estimation during static noise estimation to provide better efficiency [39, 41, 45, 87], and multiplies the noise internally to ensure enough noise bits suggested in [76]. Recently, Guo *et al.* [55] show that relying on non-worst-case noise estimation undermines noise-flooding countermeasures for achieving IND-CPA^*D*^ and implement a key-recovery attack on OpenFHE. In their attack, the adversary has the freedom to select a different evaluation function for noise estimation; however, in our application, the senders are semi-honest, who run the noise estimation, and add the correct amount of noise using worst-case noise estimation using [Table 1, [76]]. Hence, this attack does not apply to our protocol. Alexandru *et al.* [5] have also pointed out that these attacks are the result of misusing the OpenFHE library by employing different (incompatible) circuits during noise estimation.

#### 4.7.4 Tradeoffs.

The process of applying a DEP followed by Chebyshev approximation requires significant depth, but this depth does not depend on the number of senders involved. Each sender can perform this computation in parallel, but this step may be expensive. Thus, even though we require a higher computational complexity compared to other methods for a small number of senders, this complexity does not depend on the number of senders and stays moderately low for a significantly higher number of senders. The protocol’s computation and FHE multiplicative depth only depend on the size of the sender’s set and not the number of senders. We also note that FHE batching can only be applied to either the receiver or senders, but not both. Applying batching to the receiver’s elements will require using hashing techniques (e.g., cuckoo hashing), which will eventually require pre-processing of the elements.

## 5 CHALLENGES

### 5.1 Increasing Throughput

Using our PSMT protocol, senders encode each of its sets’ elements into separate plaintexts and individually compute the polynomial approximation. A single query can take time on the order of seconds, highlighting the need for better throughput. The throughput of our PSMT protocol can be improved using the SIMD feature available in FHE schemes. The CKKS scheme supports the packing of multiple message vectors into a single ciphertext where the slots of the ciphertexts hold different values. This allows slot-wise addition and multiplication [16, 50, 52, 101]. The CKKS scheme can encrypt η=N/2 elements in R into a single ciphertext using this mapping. The batching technique allows the sender to operate on η items simultaneously, resulting in η-fold improvement in both the computation and communication. To enable batching when not all slots are used, we fill the remaining slots with a dummy value.

### 5.2 Supporting Larger Sets

Increasing the capacity of the protocol to support large senders’ sets requires the use of larger plaintext spaces. In prior work on FHE-based PSI [21, 23, 38], the sender partitions their elements into disjoint sets and computes the intersection of receiver elements with each partition. This can reduce the effective set size in each computation, but partitioning increases the number of results to be sent back. Although the results can be multiplicatively aggregated, it requires more depth. An advantage of our design is that we gain the ability to partition computations and thereafter aggregate results from the partitions nearly for free.

Our protocol can be extended to use very large sender sets. However, we note that it will require more depth and computing power due to the large FHE parameters required for the DEP procedure and Chebyshev approximation. Since our final result is the summation of individual results from the senders, increasing the senders’ set sizes does not dramatically increase the communication size.

### 5.3 Reducing False Positives

In the existing works, false positives occur due to hashing collisions [23] or representation of the set elements using some probabilistic encoding techniques like bloom filter encoding [99]. In our protocol, false positives can occur due to bad approximation accuracy of the VAF. After applying the DEP procedure, we found that the homomorphic approximation for the VAF was not accurate enough within the range [−3, 3]. While the approximation would correctly map zero values to K, the non-zero values in the range [−3, 3] would be mapped to values far too close to K. Hence, instead of producing a hump at 0, we observed that the approximation would produce a flatter curve. These values would later contaminate the summation outcome, leading to false positives. To solve this issue, we used a technique involving homomorphic squaring and scaling. Homomorphically squaring the values in the range (−1, 1) would map them to smaller and smaller values. Then, we would scale them by a scaling factor ρ∈(2,2.8) so they remain within (−1, 1). Finally, we apply homomorphic squaring again, which would square ρ for intersection and augment the difference between values mapped to zero and non-zero. After using the aforementioned techniques, κ is the highest value approximated by the Chebyshev (see Table 5) when ${diff}_{i}$ is at its minimum value of one.

Our complete protocol after incorporating the aforementioned solutions is detailed in Figure 4.

## 6 EVALUATION

We implemented our proposed PSMT protocol using C++17 and OpenFHE v1.0.3 [2]. The anonymized source code is available at https://anonymous.4open.science/r/psmt-7777. We used the threshold variant of CKKS scheme [29] and employed the default FHE parameters provided in OpenFHE using [3] and the noise smudging parameters provided in [76] to ensure a (128, 36)-bit computational and statistical security. To be able to support a higher amount of noise bits during noise smudging, we recompiled OpenFHE with NATIVE_SIZE = 128 flag. Our experiments were run on a server with an AMD EPYC 7313P processor and 128GB of memory, running Ubuntu 20.04. In our LAN setup, we assumed a 10 Gbps bandwidth and 0.2ms round trip time (RTT) latency, while our WAN assumed 200 Mbps and 1 Gbps bandwidths with an 80ms RTT latency.

We evaluate the performance in terms of computational and communication costs. For computational latency, we evaluate per-sender query runtime and the runtime for aggregating results from multiple senders by performing 5 runs for each combination of parameters and taking the average. The bit-length δ of the set elements is matched to the plaintext space accommodated by the DEP and Chebyshev parameters. The senders’ individual computations are independent in our protocol. We assume that after receiving the query ciphertext from the receiver, each sender operates on their own set in parallel. We choose the number of senders for every senders’ set size from 2^7^ to 2^25^ such that each sender has to evaluate a single ciphertext, i.e., a single sender possesses at most η elements. In case we have fewer senders for a given |X|, which results in a single sender possessing more than η elements, we assume that senders pack their set elements in multiple ciphertexts and evaluate them parallelly and homomorphically summates them to a single ciphertext. To obtain the noise for noise smudging, we assume senders perform static noise estimation offline using the publicly available input bounds reflecting actual data and the VAF function to be evaluated. This is a one-time estimation process that solely depends on the query computation time and induces negligible latency while considering PSMT for a large number of senders. To better understand the scalability of our protocol, we evaluate it on the range of the number parties $l=2^{n}$ where n∈[2,12].

### Baselines.

Many variants of PSI, MPSI, and PMT protocols are designed to handle specific or more general scenarios. Selecting a specific protocol for comparison with our work is a complex task, given our novel privacy model with server-side encryption, provenance privacy, and a high number of senders. Considering the existing works that we discussed in Section 2, [71], [7], [97], [94] and [18] either lack scalability for a large number of senders, do not provide a public implementation or do not support multiple senders. ORAM-based approaches such as [104] simply do not support a higher number of senders for comparison with our work. [23] is an older work and serves as the foundation for [21] and [38]. Thus, we primarily compare our protocol to the following state-of-the-art baseline FHE-based and non-FHE-based PSI and MPSI protocols: Cong *et al.*, [38], Bay *et al.* [8] and Nevo *et al.* [84]. Cong *et al.*’s protocol uses the BFV FHE scheme [47] and for a fair comparison, we implemented their protocol for a multiparty setting using a threshold variant of BFV in OpenFHE by using their public implementation [1], originally implemented in SEAL [22]. We used the default noise-flooding mechanism in OpenFHE for BFV to ensure IND-CPA^*D*^ in the threshold setting. Similarly, for non-FHE-based protocols we ran the public implementations (both in C++) of the MPSI protocols of Bay *et al.* [8], which is one of the fastest-known MPSI protocols for smaller set sizes and a large number of parties and Nevo *et al.* [84], which is one of the most scalable maliciously secure MPSI protocol based on OPPRF and OKVS on our server for performance comparison.

**Figure 6:**
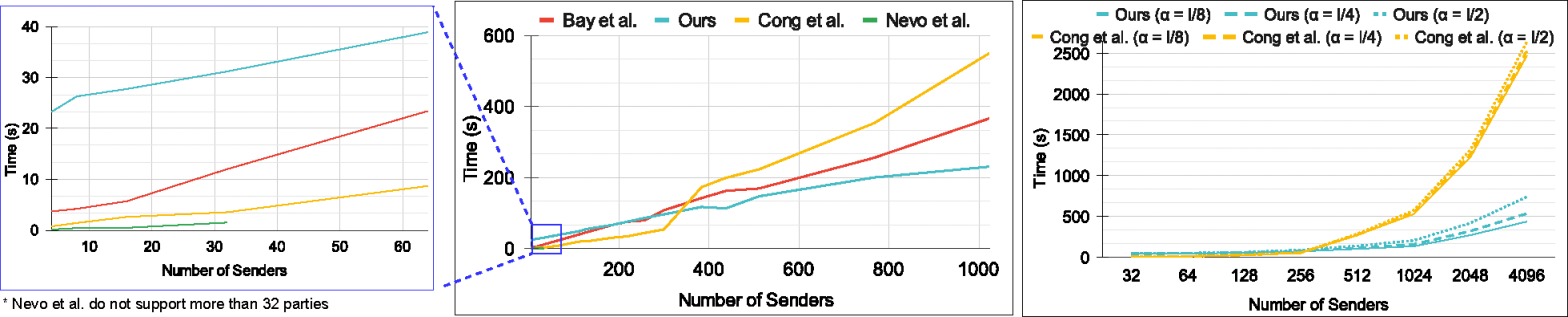
**Runtime comparison for Bay *et al.* [8] and Nevo *et al.* [84] with ours and Cong *et al.* [38] (middle, zoomed-left). Total computation time comparison for Cong *et al.* [38] vs. our protocol for** l<4096 **and** $\alpha \in \{\frac{l}{8},\frac{l}{4},\frac{l}{2}\}$**; senders’ set size is set to** 2^15^ **(right).**

Table 3 shows the overall communication overhead and computation time, which is the total time required to complete the DEP and Chebyshev approximation using the parameters in Table 5. Observe that the size of messages from the receiver to senders increases with senders’ set sizes due to larger senders’ set size requiring query ciphertexts, which should be able to tolerate a higher amount of noise and, hence, higher FHE depth for the DEP and Chebyshev. The inter-sender communication is largely determined by the size of ciphertexts sent by senders to the leader after query computation. The leader sender has to then send back α aggregated ciphertexts for partial decryption. Since the aggregated ciphertext’s size is significantly smaller, the communication is not affected much by higher values of α in our protocol (Figure 6, right). Finally, the message size from the senders to the receiver depends on α, and it remains very low as it only contains the summation result ciphertext.

For various settings of |𝒳|, we set the highest number of parties to 1024 (modest for most applications discussed in Section 1) and examine the aggregation latency and communication overhead. Most related state-of-the-art works are only evaluated for a small number of senders (usually 100 or less) [8, 9, 71]. The aggregation time (not counting encryption and partial/final decryption) of our protocol stays within 100 seconds for $|𝒳|\leq 2^{25}$, and our protocol can be easily extended for a higher number of senders (up to 4096 in Figures 6 and 8). Moreover, the corruption threshold α does not significantly affect the computational or communication overhead. For the applications discussed in Section 1, we can observe that our protocol can handle the increasing number of parties very efficiently. The parties do not need to store any auxiliary data for computing PSMT. Moreover, parties in these applications (e.g., government agencies, banks, insurance companies) are often equipped with high bandwidth networks and connected through LANs, which helps minimize the communication latency in our protocol. We note that we only report computational latency for only up to 4096 parties, but this is only due to memory constraints when running the protocol with a huge number of senders in a single system.

### Comparison with FHE-based protocol.

**Figure 7:**
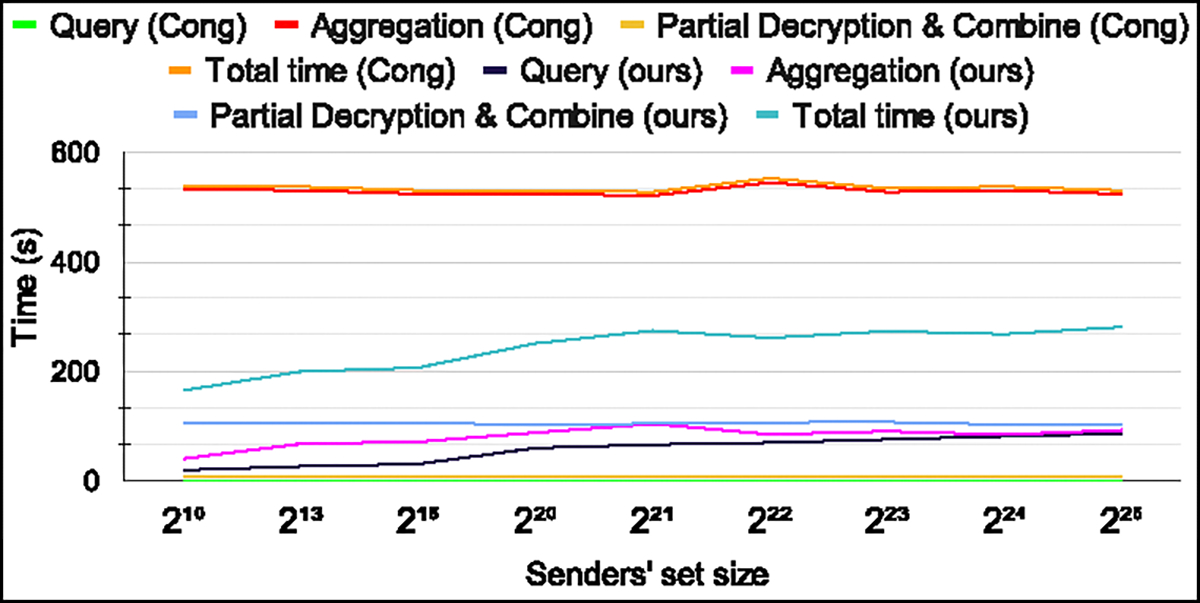
Computation time comparison for Cong *et al.* [38] vs ours for different senders’ set size. Number of senders is set to 1024. **Bay *et al.* and Nevo *et al.* were omitted due to inefficiency for larger set sizes and a high number of senders, respectively.

**Figure 8:**
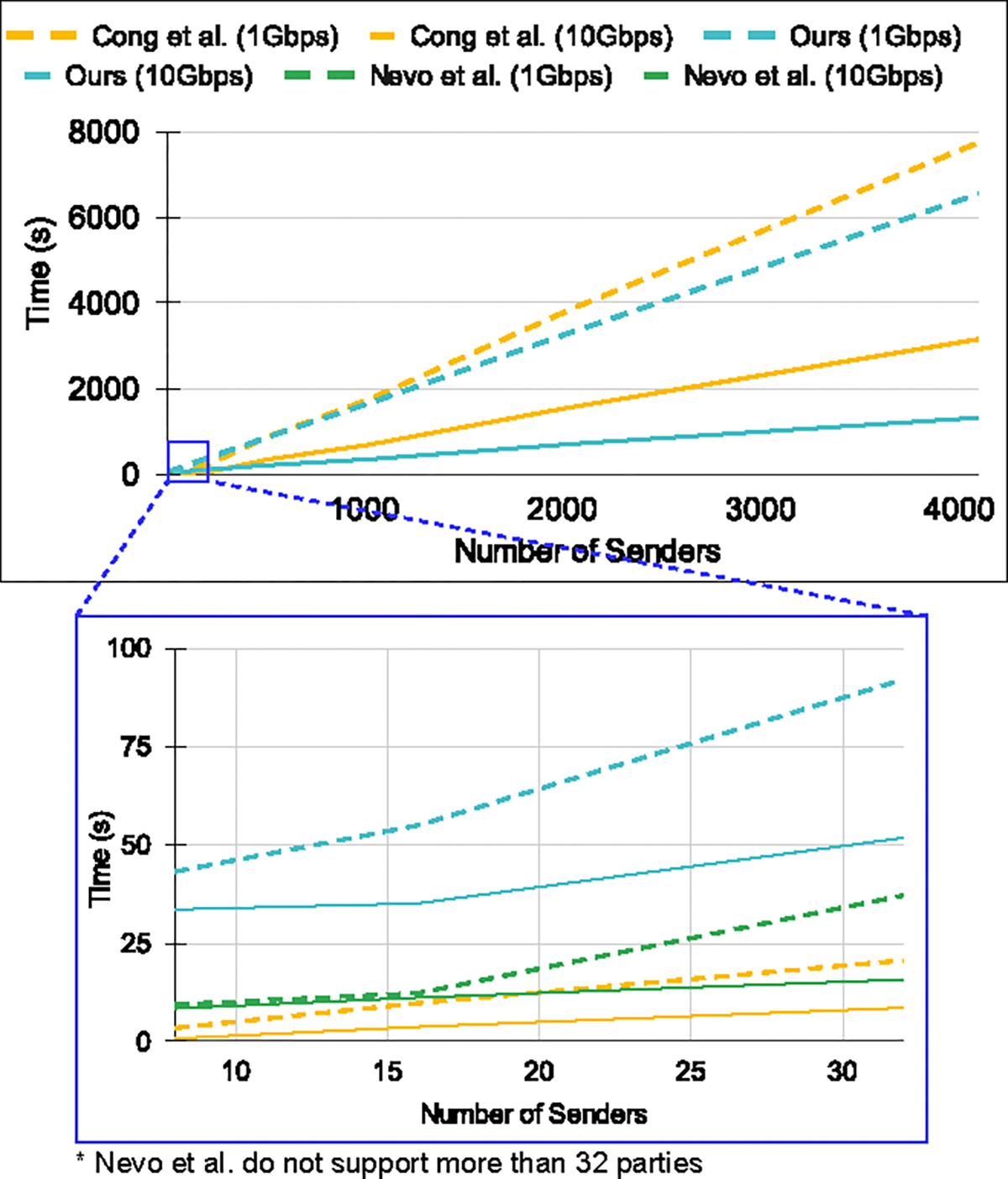
**Total runtime comparison for Cong *et al.* [38] and Nevo *et al.* [84] with ours. Senders’ set size is set to** 2^15^ **and** $\alpha =\frac{l}{2}$. **Bay *et al.* omitted as they do not support 2^15^ **set size.**

We compare the computational latency of our protocol and Cong *et al.*’s protocol [38] in the multi-party setting in Figures 6 to 8. For the number of senders less than about 320, [38] is faster than our protocol, but for higher number of senders, our protocol outperforms [38]. As the number of senders increases, aggregation time for [38] becomes the bottleneck due to *multiplicative* aggregation. The query computation time for our protocol is slower than [38]. Similarly, partial decryption time and ciphertext combining time are also slower for our protocol, as these operations are inherently slower in CKKS compared to BFV. However, our protocol is almost 3.3 – 5.6× faster for various values of α in Figure 6 (right), and |𝒳| in Figure 7 when the aggregation time is also taken into account for the overall latency. We observe this speedup due to our *summation* based approach for aggregation. Moreover, continuing to increase the number of senders results in an even greater computational latency benefit.

In terms of communication size, [38] has an advantage over our protocol since it uses smaller FHE parameters while query computations as they operate on unencrypted datasets (Table 4). We, however, process fully encrypted datasets and provide security against the senders that necessitate the use of non-scaler homomorphic operations requiring relatively larger FHE parameters. Moreover, our communication cost scales much better when dealing with a high number of senders, as depicted in Figure 8. Our communication is mainly dependent on |𝒳| and grows slowly with the increasing number of senders. Considering $l\in \left[2^{9},2^{13}\right]$ and high bandwidth network environments (around 1Gbps or more), our total runtime latency is up to 5.6× better than [38].

### Comparison with non-FHE-based protocols.

For non-FHE-based protocols, multiplicative depth is not a significant issue, but most of these protocols have high communication and no post-quantum security. These protocols either support a large senders’ set size or a large number of senders but not both. We compared both our work and [38] to Bay *et al.* [8] and Nevo *et al.* [84] in the MPSI setting with $\alpha =\frac{l}{2}$ collusion threshold.

In terms of communication, [8] require five rounds while ours and [84] need four. [8] only provide a theoretical level communicational analysis, lacking an implementation level evaluation, thus, for communication we only compare ours with [38] and [84]. Theoretically, clients in [8] send encrypted bloom filters comprising m ciphertexts, where m is determined by the number of hash functions h and the dataset size n. In experiments, [8] set h=7 and $m=\lfloor \frac{7n}{log2}\rfloor$ bits to achieve a 1% false positive rate, while our method maintains a negligible false positive rate. For sensitive applications requiring a much lower false positive rate, [8] must use higher m and h, which will incur significantly larger overheads.

[8, 38, 84] access the senders’ set elements in plain for encoding while we operate on encrypted sets. In [8], senders can have duplicate elements among each other, but in ours, we assume that all senders have distinct elements. Senders’ set size is set to 2^7^ for [8], but for the number of parties higher than 128, 256, and 512, we set the senders’ set size to 2^8^, 2^9^, and 2^10^ for us and [38], [84] respectively even though it favors [8]. We report the runtime comparison using these settings in Figure 6 (middle, left-zoomed).

[84] are extremely fast as they employ very efficient OPPRF and OKVS-based primitives; however, they can only handle up to 32 parties. For applications involving a limited number of parties and malicious security, [84] is preferable. [38] and [8] are better for applications having less than around 300 parties but [8] are limited in set size. Our protocol scales much better and is up to 1.5× and 2.4× faster than [8] and [38] for up to 2^10^ senders. Hence, ours is preferable for applications with a very high number of parties that require security against the senders.

### Communication and total runtime.

We compare our protocol to [84] and [38] in Table 4 for communication size. We limit l=15 as [84] only provide communication analysis for up to 15 parties in [[84], Table 4]. Our protocol outperforms [84] when the collusion level reaches α=l−1, though [84] excels at lower α values. [38] surpasses both our method and [84] in terms of communication only. In Figure 8, we show the total runtime latency of our protocol compared to [38] and [84]. Even though [38] and [84] have lower latencies for a smaller number of parties (due to unencrypted senders’ sets), ours is up to 2.4× faster for a higher number of parties as we rely on *summation*-based aggregation.

### Comparison with other works.

[71] is close to our work in the MPSI setting; however, it scales poorly for a large number of parties, taking almost 300 seconds of computation time for 15 parties. [9] provide a maliciously secure MPSI based on garbled bloom filters and k-out-of-N OT. [9] and [71] both has been compared against [84] hence we do not compare against [9] and [71]. [24] employ a multi-query reverse private membership test protocol, but their implementation is not compatible with multiple parties. [112] achieve maliciously secure MPSI using bloom filters for large inputs; however, they lack a public implementation for comparison.

## 7 CONCLUSION

In this work, we introduce the concept of a Private Segmented Membership Test (PSMT) for cases where users wish to query a set held segmented among many data holders while ensuring provenance privacy. We show a basic protocol to solve PSMT based on approximate-arithmetic threshold FHE and provide details about overcoming various technical challenges to make our solution feasible. We further guarantee IND-CPA^*D*^ security for threshold CKKS using noise-smudging techniques. Our experiment shows the scalability of our protocol that aggregates the penultimate results from data holders. In future work, we aim to explore new avenues for further optimizations, discussions, and better communication.

## Figures and Tables

**Table 1:** Comparison of existing works to our work.

Protocol	Construction	Class	Post-Quant.	S.A.S	L.F.P.	Agg. Comp.	Collusion	Rounds	Adversary Model

Chen *et al.* [23]	FHE	PSI	✓	×	✓	$𝒪\left(\log\;l\right)$	–	2	Semi-honest
Chen *et al.* [21]	FHE, OPRF	PSI	×	×	✓	$𝒪\left(\log\;l\right)$	–	2	Malicious
Cong *et al.* [38]	FHE, OPRF	PSI	×	×	✓	$𝒪\left(\log\;l\right)$	–	2	Malicious
Kolesnikov *et al.* [71]	OPPRF	MPSI	×	×	✓	–	*α < l*	4	Semi-honest
Ramezanian *et al.* [97]	Bloom/Cuckoo Filter, HE	PMT	×	×	×	–	–	2	Semi-honest
Pinkas *et al.* [94]	Oblivious Transfer	PSI	×	×	✓	–	–	2	Semi-honest
Bay *et al.* [8]	Bloom Filter	MPSI	×	×	×	–	*α < l*	5	Semi-honest
Nevo *et al.* [84]	OPPRF, OKVS	MPSI	×	×	✓	–	*a < l*	4	Malicious
Vadapalli *et al.* [104]	DPF	ORAM	×	✓	✓	–	–	log(*l*) + 1	Semi-honest

This work	Threshold FHE	PSMT	✓	✓	✓	$𝒪\left(1\right)$	*α* < *l*/2	4	Semi-honest

Notation: *l* parties; *α* parties are corrupted and colluding; S.A.S: Security against the senders; L.F.P.: Low false positive rate (less than 10^−3^); Agg. Comp.: Multiplicative aggregation overhead of FHE; OPRF, OPPRF, DFP, and HE denote Oblivious Pseudorandom Function, Oblivious Programmable Pseudorandom Function, Distributed Point Function, and Homomorphic Encryption, respectively. Post-Quant. refers to the security against quantum computer-capable adversaries; – denotes not applicable.

**Table 2:** List of notations and descriptions

Notation	Description

*l*	The total number of parties (*l* – 1 senders)
*X*_*i*_	Set of the *i*^th^ sender (owned by the data-owner)
*X*_*l*–1_	Senders’ leader
𝒳	Union of the senders’ sets
*δ*	The length of the bit-string
$c_{x_{i}}$	Ciphertext of the *i*^th^ sender
$c_{y}$	Ciphertext of the receiver

*N*	The ring dimension in FHE (power of 2)
*q*	Ciphertext modulus
*D*	The FHE multiplicative depth
*η*	The batch size of FHE scheme
*α*	Number of secret-key shares
*σ*	Standard deviation of smudging noise
*a*	Number of adversarial queries
*s*	Statistical security bits
*λ*	Computational security bits
*D_R,σ_*	Discrete Gaussian noise distribution

*L*	Lower bound of interval for DEP
*R*	Upper bound of interval for DEP
*n*	Number of iterations for DEP
*c*	Degree of polynomial for Chebyshev approximation
*j*	Count of 1^*st*^ homomorphic square operation
*k*	Count of 2^*nd*^ homomorphic square operation
*ρ*	Scaling factor for reducing false positives
*τ*	Threshold required to confirm an intersection
*κ*	Limit for the random mask
${\mathrm{diff}}_{i}$	FHE-ciphertext (vector denoting $c_{x_{i}}-c_{y}$)
${\mathrm{etan}}_{i}\;()$	A piecewise function that takes ${\mathrm{diff}}_{i}$ as input
*K*	Output of ${\mathrm{etan}}_{i}\;()$ when input is 0 (maxima of VAF)
*S*	Parameter for controlling input range of 0 in VAF

**Table 3:** Communication and computation overhead in our protocol. Agg. denotes the total time required to aggregate the ciphertexts. R-to-S, S-to-S, and S-to-R denotes receiver to sender, inter-sender, and sender to receiver, respectively. Query refers to the query computation time for each sender.

|𝒳|	Message size (MB)			No. of Senders	Agg. (second)	Query (second)	Depth
	R-to-S	S-to-S	S-to-R				

2^7^	45	49.1	2.1	128	6.53	13.20	21
2^8^	49	53.1	2.1	256	14.12	15.24	23
2^10^	63	67.1	2.1	1024	40.29	19.09	30
2^13^	79	83.1	2.1	1024	67.51	26.00	38
2^15^	87	91.1	2.1	1024	71.16	30.07	42
2^20^	107	111.1	2.1	1024	87.78	59.67	52
2^21^	111	115.1	2.1	1024	103.98	64.65	54
2^22^	115	119.1	2.1	1024	84.63	68.90	56
2^23^	119	123.1	2.1	1024	90.34	75.80	58
2^24^	123	127.1	2.1	1024	84.09	81.08	60
2^25^	127	131.1	2.1	1024	92.53	86.76	62

**Table 4:** **Communication cost for our protocol compared to Cong *et al.* [**38**] and Nevo *et al.* [**84**] for up to** *l* = 15, *α* = *l* – 1 **and** 2^20^ **senders’ set size. “Total Comm. size” refers to the communication size of sent/received data between all parties**^[1,2]^. **Bay *et al.* [8**] omitted as they lack an implementation level communication analysis and cannot handle** 2^20^ **senders’ set size.**

Params.		Total Comm. size			Comm. time (seconds)								

*l*	*α*	[38]	[84]	Ours	10 Gbps			1 Gbps			200 Mbps		

					[38]	[84]	Ours	[38]	[84]	Ours	[38]	[84]	Ours

4	1	180.1	201.3	759.3	0.5	0.2	0.6	1.8	1.6	6.1	7.5	9.0	30.7
	3	195.5	2225.6	771.7	0.5	1.8	0.6	1.9	17.8	6.2	8.1	89.3	31.2

10	1	497.2	453.0	2043.3	0.7	0.4	1.6	4.3	3.6	16.3	20.2	18.4	82.1
	4	515.8	1054.6	2061.9	0.7	0.8	1.7	4.5	8.4	16.5	21.1	42.5	82.8
	9	546.8	5563.9	2082.9	0.8	4.5	1.7	4.7	44.5	16.7	22.2	222.9	84.0

15	1	742.7	662.7	3113.3	0.9	0.5	2.5	6.3	5.3	24.9	30.0	26.8	124.9
	4	761.3	1254.6	3131.9	0.9	1.0	2.5	6.4	10.0	25.1	30.8	50.5	125.6
	7	779.9	1416.9	3150.5	1.0	1.1	2.5	6.6	11.3	25.2	31.5	57.0	126.3
	14	823.3	8345.9	3193.6	1.0	6.7	2.6	6.9	66.8	25.6	33.3	334.2	128.1

1 [38] and [84] do not provide security of datasets against the senders.

2 [84] do not support sender side encryption. Implementing sender side encryption in [38] results in impractical runtimes (multiple hours or days) due to huge overheads for optimization operations and intersection polynomial generation for interpolation in the homomorphic domain.

## Footnotes

1 Some guidelines for choosing parameters for polynomial approximation can be found at https://github.com/openfheorg/openfhe-development/blob/main/src/pke/examples/FUNCTION_EVALUATION.md

## A PROTOCOL PARAMETERS

We analyzed various levels of parameters for the DEP procedure and Chebyshev-based approximation and how they affect the performance and accuracy of our protocol for PSMT. Using $B(x)=x-\frac{4}{27}x^{3}$, it is imperative to maintain L below $1.5\times \sqrt{3}$ while using Domain Extension Polynimials [32]. Thus, for expanding the domain range, it requires increasing R and n so that $\left[-L^{n}R,L^{n}R\right]$ adequately accommodates large input sets without amplifying errors and computation. However, using arbitrary large values for R and n would require very high depths for homomorphic computation and amplify errors in the approximation. Using smaller values of L, especially close to 1.5, greatly increases the accuracy of the DEP method, but this results in larger values for other parameters, incurring very high computational costs.

The optimal DEP, Chebyshev, and optimization parameters for running our protocol for various senders’ set sizes are provided in Table 5. K and S are set to 1 and 10, respectively, for the VAF. |X| denotes the size of the senders’s set. “Non-intersection” denotes the summation value of ciphertext’s slots when there is no intersection, and “Intersection” similarly denotes the summation value of ciphertext’s slots during an intersection. To confirm an intersection for l<4096, setting threshold τ>200 is sufficient for the parameters described in Table 5. A higher value of τ handles the precision loss occurring due to the additional added noise from noise-smudging in cases where $z=\sum_{i=1}^{l-1}{etan}_{i}$ overflow beyond negligible values. The value τ can be adjusted to higher values for a higher number of parties. We note that the parameters we provide are highly optimized for the particular computations, and some values in Table 5 were found empirically.

**Table 5:** Our protocol parameters to solve PSMT for various senders’ set sizes

|𝒳|	DEP			Chebyshev Parameters		Optimization			Aggregation Result	
	L	R	n	*c*	*κ*	*j*	*k*	*ρ*	Non-intersection	Intersection

2^7^	2.50	21.0	2	27	3.4 × 10^−5^	3	3	2.5	3.1 × 10^−5^	1528.18
2^8^	2.56	16.0	3	27	3.2 × 10^−1^	1	3	2.5	2.9 × 10^−1^	1526.54
2^10^	2.58	24.0	4	27	1.8 × 10^−6^	4	3	2.5	1.1 × 10^−4^	1525.88
2^13^	2.58	27.5	6	27	5.6 × 10^−3^	4	3	2.5	4.7 × 10^−3^	1525.88
2^15^	2.58	43.5	7	27	3.7 × 10^−1^	4	3	2.5	4.9 × 10^−1^	1526.63
2^20^	2.59	200.0	9	247	1.2 × 10^−1^	2	3	2.5	2.2 × 10^−1^	1526.11
2^21^	2.59	400.0	9	247	7.3 × 10^−1^	4	3	2.7	8.1 × 10^−1^	2824.66
2^22^	2.59	800.0	9	247	7.6 × 10^−1^	6	3	2.7	8.8 × 10^−1^	2824.29
2^23^	2.59	1600.0	9	247	6.3 × 10^−1^	8	3	2.7	9.1 × 10^−1^	2823.91
2^24^	2.59	3200.0	9	247	7.9 × 10^−1^	10	3	2.7	8.8 × 10^−1^	2824.20
2^25^	2.59	6400.0	9	247	2.7 × 10^−1^	12	3	2.7	7.4 × 10^−1^	2823.92
